# In Vivo Characterization and Tissue Tropism of a Wild-Type Yellow Fever Virus Isolate from the 2017–2018 Brazilian Outbreak in C57BL/6 IFNAR1^−/−^ Mice

**DOI:** 10.3390/v17101325

**Published:** 2025-09-29

**Authors:** Ana Luiza Campos Cruz, Natália Lima Pessoa, Ester Maria Paiva Silva, Sabrynna Brito Oliveira, Jéssica Pauline Coelho Souza, Samantha Stephany Fiuza Meneses Viegas, Anna Catarina Dias Soares Guimarães, Pedro Augusto Alves, Cintia Lopes de Brito Magalhães, Thomas P. Monath, Olindo Assis Martins-Filho, Andréa Teixeira-Carvalho, A. Desiree LaBeaud, Nidia Esther Colquehuanca Arias, Betânia Paiva Drumond

**Affiliations:** 1 Laboratório de Vírus—Institute of Biological Sciences (ICB), Federal University of Minas Gerais (UFMG), Belo Horizonte 31270-901, MG, Brazil; natalinha_lima@hotmail.com (N.L.P.); esterpaiva19042001@gmail.com (E.M.P.S.); sabrynnabrito@gmail.com (S.B.O.); jescoelhobio@gmail.com (J.P.C.S.); samfviegass@gmail.com (S.S.F.M.V.); annacatarinaanna@gmail.com (A.C.D.S.G.); 2 Oswaldo Cruz Foundation (FIOCRUZ)—René Rachou Institute, Belo Horizonte 30190-002, MG, Brazil; pedro.alves@fiocruz.br (P.A.A.); atcteixeira@gmail.com (A.T.-C.); 3 Crozet BioPharma LLC, Lexington, MA 02421, USA; cintiamagalhaes@gmail.com (C.L.d.B.M.); tom@quigleybio.com (T.P.M.); 4 Department of Pediatrics, Division of Infectious Diseases, Stanford University School of Medicine, Stanford, CA 94305, USA; dlabeaud@stanford.edu

**Keywords:** yellow fever, wild-type YF, brazilian isolate, C57BL/6 IFNAR1^−/−^ mice, male, female

## Abstract

Yellow fever remains a significant public health concern in endemic regions of South America and Africa, where periodic outbreaks continue to challenge surveillance and control efforts. Despite the widespread use of vaccines and historical YFV strains in experimental settings, there is limited information on the pathogenic behavior of contemporary wild-type isolates in animal models. To address this gap, this study aimed to develop and characterize a murine model infected with a wild-type YFV strain isolated in 2018, from Brazil’s largest sylvatic outbreak in decades. In this study, four-week-old male and female C57BL/6 IFNAR1^−/−^ mice were subcutaneously infected with WT YFV. Mice exhibited a nearly 50% survival rate and developed several clinical signs. Viral loads were assessed in serum and some tissues, collected either upon euthanasia of moribund animals or at the end point. YFV RNA was detected in all sampled tissues and serum. Infectious viral particles were identified in the brains of both sexes and in the testis. No statistically significant differences were observed between males and females in survival, clinical signs, or viral loads. Altogether, this study provides a robust and reproducible murine model for wild-type YFV infection, offering a valuable platform for investigating viral pathogenesis, host responses, and potential therapeutic interventions.

## 1. Introduction

Yellow fever (YF) is a disease caused by the yellow fever virus (YFV), which is transmitted through mosquito bites to humans and non-human primates (NHPs) [1,2]. YFV (*Orthoflavivirus flavi, Flaviviridae* family) [3] is endemic to both the Americas and Africa. In South American countries, between 1974 and 2023, 7921 cases were notified, the vast majority from Brazil [4,5]. In Africa, 38,261 cases were reported between 1974 and 2023, most of which occurred in West Africa, followed by Central, East, and Southern Africa [6].

Currently, in South America, only the sylvatic transmission cycle is reported [1]. Urban transmission in Brazil was last reported in 1942 [7], reinforcing the importance of monitoring sylvatic cycles. YFV is considered endemic in the Amazon basin, with periodic outbreaks of sylvatic YF reported in other regions of the country [2]. Beginning at the end of 2016, Brazil experienced its most significant outbreak of sylvatic YF since the eradication of urban transmission [2]. Between 2016 and 2022, 2290 cases and 783 deaths were confirmed, which is almost twice as many cases as were recorded in the previous 55-year period (1960 to 2015) [4]. This outbreak was driven by a new lineage of YFV belonging to the South American genotype I [8,9,10,11,12]. This lineage harbored previously undescribed amino acid substitutions [8,9,11], some of which occurred near structural domains of viral proteins [11], potentially affecting the virus’s infectivity [8].

The pathogenesis of YF involves a complex interplay between viral replication, host immune responses, and tissue-specific tropism. Following the inoculation of the vertebrate host via mosquito bite, YFV first infects dendritic cells, which then migrate to lymph nodes. In these organs, the virus replicates and enters the bloodstream, disseminating to various tissues. In humans, YFV causes mainly viscerotropic disease, and the liver is the primary site of YFV replication. However, the virus also shows tropism for cells in the heart, kidneys, vascular endothelium, and other organs [13,14]. Understanding the pathogenesis of YF remains critical, particularly considering the emergence of new viral lineages with unique genetic and phenotypic characteristics that may influence virulence and transmission dynamics, as observed recently in Brazil [8]. Understanding YF pathogenesis is essential, especially in light of the emergence of novel viral lineages, such as those recently identified in Brazil, that carry distinct genetic features potentially influencing virulence and transmission dynamics [8].

To unravel the mechanisms of YFV pathogenesis and advance the development of antivirals and vaccines, robust and reliable animal models are indispensable. Among available options, mice stand out as a preferred experimental model due to their well-characterized immune system, genetic tractability, and extensive use in biomedical research. However, adult mice are resistant to peripheral YFV infection, primarily due to the production of type I interferon (IFN-I) [15]. To overcome this limitation, IFN receptor knockout mice can be employed. A129 mice, which are deficient in the IFN-α/β receptor (IFNAR1^−/−^), have been shown to be susceptible to peripheral infection with wild-type (WT) YFV strains, such as Asibi and Angola73. Another widely used mouse strain in research is C57BL/6. However, a reliable model for peripheral infection with WT YFV has not yet been established for this mouse lineage. Furthermore, most studies have used either vaccine strains or the historical Asibi strain (isolated in 1927) [16]. This highlights a critical need to establish a murine infection model based on a contemporary Brazilian WT YFV isolate from a lineage associated with a recent large-scale outbreak, to more accurately reflect current viral behavior and pathogenesis. Therefore, this study aimed to establish a murine infection model using C57BL/6 IFNAR1^−/−^ mice infected with a wild-type YFV strain isolated from a human case during the 2018 outbreak in Brazil, a YFV lineage associated with the country’s largest sylvatic epidemic in decades. Both C57BL/6 IFNAR1^−/−^ and A129 IFNAR1^−/−^ mice lack the type I interferon (IFN) receptor, rendering them highly susceptible to viral infections. We chose the C57BL/6 background in this work due to its broad availability in our facility, its extensive use as a genetic background in experimental models, and the advantage of enabling future studies that may require access to the large repertoire of transgenic and knockout strains already established on the C57BL/6 background. This model proved to be robust and reproducible, allowing detailed investigation of viral tissue tropism, clinical progression, and potential sex-related differences under controlled experimental conditions.

## 2. Materials and Methods

### 2.1. Cell Line and Virus

Vero cells (CCL-81), obtained from the American Type Culture Collection (ATCC, Manassas, VA, USA), were cultured in Minimum Essential Medium (MEM; Cultilab, São Paulo, Brazil) supplemented with 5% fetal bovine serum (FBS; Cultilab, São Paulo, Brazil) and antibiotics: Penicillin (Benzilpen^®^, Rio de Janeiro, Brazil 100 IU/mL), streptomycin (Sigma-Aldrich, St. Louis, MO, USA; 100 µg/mL), and amphotericin B (Cultilab, São Paulo, Brazil; 0.25 µg/mL). Cells were incubated at 37 °C in a 5% CO_2_ atmosphere.

The wild-type yellow fever virus strain YFV_HS306/2018 was originally isolated in 2018 from a patient in the acute phase of YF in the state of Minas Gerais, Brazil. The virus underwent three passages in C6/36 cells and five additional passages in Vero cells to obtain a stable clone. The working virus stock was subsequently produced in Vero cells.

### 2.2. Animal Experimentation

This study was approved by the Ethics Committee on Animal Use (CEUA) of the Federal University of Minas Gerais (UFMG), under protocol number 341/2019. All procedures were conducted in accordance with the guidelines of the National Council for the Control of Animal Experimentation (CONCEA, Brasília, Brazil).

Four-week-old male and female C57BL/6 IFNAR1^−/−^ mice were housed in Alesco microisolators (Model ALE.MIL.01.03) placed in ventilated racks (Alesco, Model ALERKD-70), with ad libitum access to food and water. The animal facility was maintained at a controlled temperature of approximately 23 °C, with a 12 h light/dark cycle. Mice were divided into groups of five and subcutaneously infected in the right hind footpad (intraplantar route) with 10 µL of viral inoculum, containing 1.6 × 10^3^ to 5.75 × 10^3^ plaque-forming units (PFU), confirmed by back titration. Mice were monitored daily for 14 days or until euthanasia, with clinical signs and body weight recorded.

Clinical manifestations were classified as mild, moderate, or severe and scored accordingly (with increasing severity receiving higher scores). Mild signs included inoculation site inflammation, piloerection, and hunched posture; moderate signs included facial swelling, conjunctivitis, penile inflammation, and tremors; severe signs included bleeding and moribund state [17] (Table S1). Mice exhibiting severe clinical signs were humanely euthanized using a lethal dose of anesthetic (300 mg/kg ketamine and 30 mg/kg xylazine), followed by cervical dislocation.

Blood and organs were collected immediately after euthanasia. Blood was obtained via cardiac puncture, transferred to microtubes, and kept at room temperature until serum separation. Organs (brain, eye, thymus, heart, lung, liver, spleen, kidney, and testis) were immediately frozen in liquid nitrogen and later stored at −70 °C. Whole organs, or portions thereof, were placed into screw-cap tubes containing three glass beads and MEM supplemented with 1% FBS, 2.5% HEPES, and antibiotics. The organ mass corresponded to 10% *w*/*v* relative to the medium volume. Tissue homogenization was performed using a bead beater (BioSpec Products, Bartlesville, OK, USA), followed by incubation at 0 °C for 3 min. Samples were then vortexed for 15 s, incubated again at 0 °C for 2 min, and centrifuged at maximum speed for 4 min at 4 °C. Supernatants were clarified by repeated centrifugation as needed until no pellets were visible.

### 2.3. YFV RNA and Infectious Loads

In some cases, a few samples needed to be diluted or processed before RNA extraction or viral titration. Given the small number of samples, sera were diluted 1:10 with phosphate-buffered saline (PBS) prior to both RNA extraction and virus titration (viral plaque assay). RNA was extracted using 140 µL of diluted sera using QIAamp Viral RNA Mini Kit (Qiagen, Hilden, Germany), following the manufacturer’s instructions.

For virus titration, a piece of each organ was weighed and placed in screw-cap tubes containing three glass beads and MEM supplemented with 1% FBS, 2.5% HEPES, and antibiotics. The medium volume corresponded to 90% (*w*/*v*), while the organ mass accounted for 10% (*w*/*v*). Samples were macerated using a bead beater (BioSpec Products, Bartlesville, OK, USA), followed by incubation at 0 °C for 3 min. Tubes were then homogenized for 15 s, incubated again at 0 °C for 2 min, and centrifuged at maximum speed for 4 min at 4 °C. Supernatants were clarified as many times as necessary until no pellets were visible in the tubes, frozen, and then used for viral plaque assays (see below).

For RNA extraction, two strategies were used. Due to the very small amount of tissue available for some organs (heart, thymus, and testis), 140 µL supernatants from macerated organs (prepared as described above) were also used for RNA extraction using QIAamp Viral RNA Mini Kit (Qiagen, Hilden, Germany), following the manufacturer’s instructions. In other cases, the available tissue quantity (liver, brain, kidney, lung, spleen, and eye) allowed RNA extraction to be performed directly from the tissue (approximately 30 mg), using the RNeasy Mini Kit (Qiagen, Hilden, Germany), also following the manufacturer’s protocol.

YFV RNA investigation was performed using primers targeting the 5′ untranslated region (UTR), as described by Domingo et al. (2012) [18]. Reactions were prepared with the GoTaq^®^ Probe 1-Step RT-qPCR System (Promega, Madison, WI, USA) using 5.0 µL Master Mix, 500 nM of each primer, 250 nM probe, 0.2 µL reverse transcriptase, 2.0 µL RNA template, and nuclease-free water to a final volume of 10.0 µL. The standard curve for absolute quantification was generated using a fragment of WT YFV, obtained from the reaction described by Domingo et al. (2012) [18], previously cloned into a pGEM plasmid and quantified using a NanoDrop spectrophotometer. Based on the plasmid quantification, the number of fragment copies was calculated. For the RT-qPCR assays, serial tenfold dilutions were prepared, ranging from 10^2^ to 10^6^ copies/μL.

YFV RNA loads were calculated based on RT-qPCR quantification relative to the tissue (serum) (Equation (1)) or tissue homogenate volume (testis, heart, and thymus) (Equation (2)) or to tissue mass (liver, kidney, brain, lung, spleen, and eye) (Equation (3)).

**Equation (1):** RNA extracted from diluted serum

(1) $$\mathrm{YFV}\;\mathrm{RNA}\;\mathrm{load}\;(\mathrm{copies}/\mathrm{mL})=\frac{\mathrm{quantity}\;\mathrm{of}\;\mathrm{RNA}\;\mathrm{copies}\;\left(\mathrm{copies}/\mu L\right)\times \;\mathrm{elution}\;\mathrm{volume}\;\mathrm{of}\;\mathrm{RNA}\;\mathrm{extraction}\;\left(\mu L\right)}{\mathrm{volume}\;\mathrm{of}\;\mathrm{serum}\;\mathrm{extracted}\;\left(\mathrm{mL}\right)\times \;\mathrm{correction}\;\mathrm{factor}}$$

where

Quantity of RNA copies = number of YFV RNA copies produced in the PCR

Volume of serum extracted = volume of serum used in the RNA extraction

Correction factor = serum volume/PBS volume

**Equation (2):** RNA extracted from supernatants/organ homogenates (testis, heart, thymus)

(2) $$\mathrm{YFV}\;\mathrm{RNA}\;\mathrm{load}\;(\mathrm{copies}/g)=\frac{\mathrm{quantity}\;\mathrm{of}\;\mathrm{RNA}\;\mathrm{copies}\;\left(\mathrm{copies}/\mu L\right)\times \;\mathrm{elution}\;\mathrm{volume}\;\mathrm{of}\;\mathrm{RNA}\;\mathrm{extraction}\;\left(\mu L\right)}{\mathrm{organ}\;\mathrm{homogenate}\;\mathrm{volume}\;\left(\mathrm{mL}\right)\times \;\mathrm{correction}\;\mathrm{factor}}$$

where

Quantity of RNA copies = number of YFV RNA copies produced in the PCR

Organ homogenate volume = volume of organ homogenate used in the RNA extraction

Correction factor = organ weight (g)/volume of MEM medium (mL) used for preparation of supernatant (organ homogenate)

**Equation (3):** RNA extracted directly from approximately 30 mg of tissues (liver, kidney, brain, lung, spleen, and eye):

(3) $$\mathrm{YFV}\;\mathrm{RNA}\;\mathrm{load}\;\left(\mathrm{copies}/g\right)\;=\;\frac{\mathrm{quantity}\;\mathrm{of}\;\mathrm{RNA}\;\mathrm{copies}\;\left(\mathrm{copies}/\mu L\right)\;\times \;\mathrm{elution}\;\mathrm{volume}\;\mathrm{of}\;\mathrm{RNA}\;\mathrm{extraction}\;\left(\mu L\right)}{\mathrm{tissue}\;\mathrm{weight}\;\left(g\right)}$$

where

Quantity of RNA copies = number of YFV RNA copies produced in the PCR

Tissue weight = tissue weight used in the RNA extraction

Viral plaque assays were performed in duplicate for each sample using Vero cells. Ten-fold serial dilutions (10^−1^ to 10^−5^) of each homogenate were prepared in MEM supplemented with 1% FBS and antibiotics. Virus adsorption occurred for 1 h at 37 °C in a 5% CO_2_ incubator, with gentle mixing every 10 min. After adsorption, approximately 1.0 mL of semisolid medium 199 containing 1% carboxymethylcellulose (CMC), 2% FBS, and antibiotics was added. Plates were incubated at 37 °C with 5% CO_2_ for 7 days. Infectious viral load was calculated based on plaque assay results relative to initial serum volume (Equation (4)) or initial organ mass used to prepare the supernatant/organ homogenate (Equation (5)).

**Equation (4):** Estimation of infectious viral load using diluted sera

(4) $$\mathrm{Infectious}\;\mathrm{viral}\;\mathrm{load}\;\left(\mathrm{PFU}/\mathrm{mL}\right)=\frac{\mathrm{titer}\;\left(\mathrm{PFU}/\mathrm{mL}\right)}{\mathrm{correction}\;\mathrm{factor}}$$

where

Titer = number of plaque-forming units obtained in the titration assay/mL of serum

Correction factor = serum volume/PBS volume

**Equation (5):** Estimation of infectious viral load using supernatant (organ homogenate)

(5) $$\mathrm{Infectious}\;\mathrm{viral}\;\mathrm{load}\;\left(\mathrm{PFU}/\mathrm{mg}\right)=\frac{\mathrm{titer}\;\left(\mathrm{PFU}/\mathrm{mL}\right)}{\mathrm{correction}\;\mathrm{factor}}$$

where

Titer = number of plaque-forming units obtained in the titration assay/mL of supernatant (organ homogenate).

Correction factor = organ weight (mg)/volume of MEM medium (mL) used for preparation of supernatant (organ homogenate).

### 2.4. Statistical Analysis

Statistical analyses were performed using GraphPad Prism 9. Survival data were analyzed using the log-rank (Mantel–Cox) test. Changes in body weight over time were analyzed using two-way ANOVA with Tukey’s post hoc test. Clinical score data were assessed using the Mann–Whitney U test, and viral load comparisons were evaluated using either the Kruskal–Wallis test or the Mann–Whitney U test, as appropriate. For viral load comparisons, statistical analyses were performed by pooling samples collected at different time points. This choice was made due to the insufficient number of animals sampled per day, which prevented robust statistical analysis restricted to same-day comparisons, a limitation of the study. Differences were considered statistically significant when *p* ≤ 0.05.

## 3. Results

### 3.1. Survival, Body Weight Variation, and Clinical Signs

**Figure 1:**
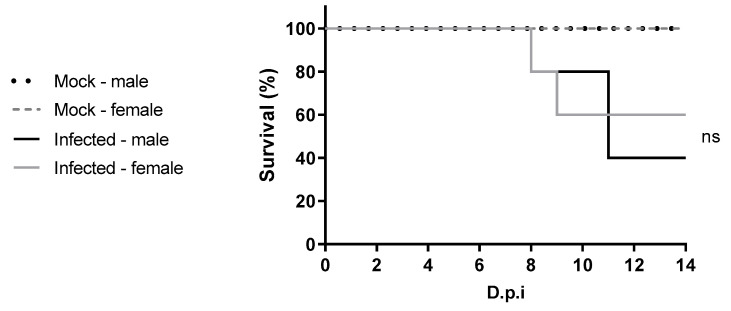
Survival of four-week-old C57BL/6 IFNAR1^−/−^ mice infected with WT YFV_HS306/2018. Male and female mice (n = 5 per group) were inoculated with 5.75 × 10^3^ PFU of virus. Mock groups received supernatant from uninfected cells. Mice were monitored daily. Statistical analysis was performed using the log-rank (Mantel–Cox) test. ns = not significant.

Four-week-old male C57BL/6 IFNAR1^−/−^ mice subcutaneously infected with 5.75 × 10^3^ PFU of YFV_HS306/2018 exhibited a mortality rate of 60%, with euthanasia performed between 8 and 11 dpi. Female mice of the same age and experimental conditions showed a mortality rate of 40%, with euthanasia occurring between 8 and 9 dpi. No statistically significant difference in lethality was observed between male and female mice (*p* = 0.0857) (Figure 1).

**Figure 2:**
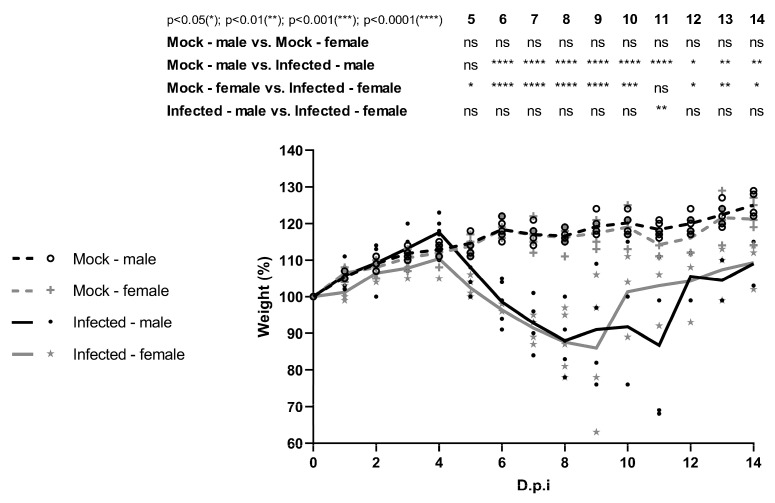
Body weight variation of four-week-old C57BL/6 IFNAR1^−/−^ mice infected with WT YFV_HS306/2018. Male and female mice (n = 5 per group) were infected subcutaneously with 5.75 × 10^3^ PFU of YFV_HS306/2018. Mock groups received supernatant from uninfected cell cultures. Body weight was recorded daily until the end of the experiment. Data are presented as mean ± standard deviation (SD). Statistical analysis was performed using two-way ANOVA with Tukey’s post hoc test. Statistical significance: *p* < 0.05 (*); *p* < 0.01 (**); *p* < 0.001 (***); *p* < 0.0001 (****); ns = not significant.

Regarding weight changes, infected females showed significant weight loss from 5 to 14 dpi (*p* < 0.05), despite gradual recovery in surviving individuals starting at 10 dpi. Infected males exhibited significant weight loss from 6 to 14 dpi (*p* < 0.05), with recovery starting on 12 dpi. Notably, on 11 dpi, infected females had significantly higher body mass compared to infected males (*p* < 0.01) (Figure 2).

Clinical signs were evaluated in infected animals (Table S1) and were absent in the mock-inoculated controls. Mild signs (inflammation at the inoculation site, piloerection, and mild hunched posture) were observed in all infected animals early during infection but were not associated with mortality when present in isolation. Moderate signs (facial edema, conjunctivitis, penile inflammation, and tremors) were noted both in animals that were euthanized and in survivors, whereas severe signs (bleeding and moribund state) were exclusively observed in animals requiring euthanasia (Figure S1).

Clinical signs appeared from 5 dpi in both sexes. Female mice exhibited mild signs (inoculation site swelling, piloerection, and hunched posture) from 5 to 12 dpi, while males showed the same signs from 5 to 14 dpi (Figure S1). Moderate signs (facial edema, conjunctivitis, and tremors) were observed in females from 6 to 10 dpi, and in males from 6 to 14 dpi (Figure S1). Notably, penile inflammation and tremors occurred concurrently in two male mice shortly before euthanasia (8–11 dpi). In both sexes, moderate signs were more pronounced in animals that were later euthanized compared to survivors (Figure S1).

Severe signs were observed in two female mice, both of which became moribund and were euthanized on 8 and 9 dpi (Figure S1). One mouse presented with a nasal hemorrhage, while the other one exhibited a cerebral hemorrhage identified during necropsy. By 13 to 14 dpi, all three surviving females were fully recovered, displaying no residual clinical signs (Figure S1).

**Figure 3:**
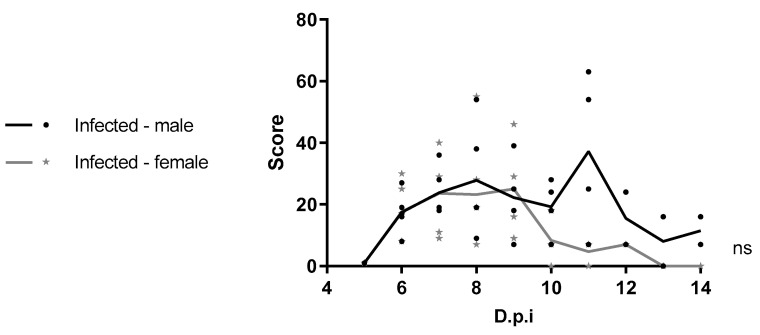
Total clinical score of observed signs in four-week-old C57BL/6 IFNAR1^−/−^ mice infected with WT YFV_HS306/2018. Male and female mice (n = 5 for each group) were infected with 5.75 × 10^3^ PFU of YFV_HS306/2018 and monitored daily for clinical signs. Mock groups were inoculated with the supernatant from uninfected cells. Results are expressed as mean ± standard deviation. Statistical analysis was performed using the Mann–Whitney test. ns: not significant.

In males, severe clinical signs (moribund state) were recorded in three animals between 8 and 11 dpi, leading to euthanasia (Figure S1). Post-mortem examination revealed various pathological findings: One male mouse showed a pale liver with hemorrhage between the eyes, another male individual presented hemorrhages in the intestine and stomach, as well as intestinal pneumatosis (air bubbles in the gut). Daily clinical scores (Figure S1) were summed for each animal. No statistically significant difference in cumulative clinical scores was observed between male and female groups (*p* = 0.1724) (Figure 3).

### 3.2. YFV RNA Loads and Infectious Viral Loads

YFV RNA loads were assessed in serum, brain, eye, thymus, heart, lung, liver, spleen, kidney, and testis. Samples were collected immediately after euthanasia from two female and three male mice that succumbed to infection: Female mice succumbed on 8 and 9 dpi and male mice on 8 (n = 1) and 11 dpi (n = 2), as well as from three female and two male mice that survived until the experimental endpoint (14 dpi).

**Figure 4:**
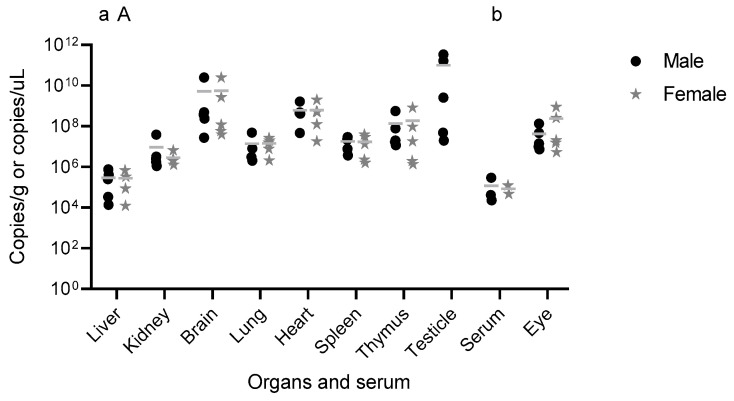
YFV RNA loads in serum and various organs of four-week-old C57BL/6 IFNAR1^−/−^ mice infected with WT YFV_HS306/2018. Male and female mice (n = 5 per group) were infected subcutaneously with 5.75 × 10^3^ PFU of YFV_HS306/2018. Following euthanasia, blood, brain, eye, thymus, heart, lungs, liver, spleen, kidneys, and testes were collected, total RNA was extracted, and YFV RNA load was assessed by RT-qPCR. Results are presented as mean values. Statistical analysis was performed using the Kruskal–Wallis test. Statistically significant differences were observed between: (a) liver vs. heart (*p* < 0.05), liver vs. brain (*p* < 0.05), liver vs. testis (*p* < 0.01) from males; (A) liver vs. brain (*p* < 0.05); (b) serum vs. testis (*p* < 0.05). PFU = plaque-forming unit.

YFV RNA was detected in serum, brain, eye, thymus, heart, lung, liver, spleen, kidney, and testis (Figure 4). YFV RNA loads ranged from 1.22 × 10^4^ to 3.37 × 10^11^ copies/g. Initially, YFV RNA loads were compared between male and female animals [female from 8 dpi (n = 2) and 14 dpi (n = 3); male from 8 dpi (n = 1), 11 dpi (n = 2), and 14 dpi (n = 2)], with no significant differences observed (*p* > 0.999999, Mann–Whitney test). Next, YFV RNA loads were compared between surviving (3 female and 2 males altogether, 14 dpi) and euthanized animals (2 female from 8 dpi; 1 male from 8 dpi and 2 males from 11 dpi altogether), with no significant differences detected across any of the tissues analyzed (*p* ≥ 0.9999, Kruskal–Wallis test). Finally, comparisons of YFV RNA loads [female from 8 dpi (n = 2) and 14 dpi (n = 3); male from 8 dpi (n = 1), 11 dpi (n = 2), and 14 dpi (n = 2)] between the different organs revealed significant differences (Kruskal–Wallis test). In males, the liver exhibited significantly lower YFV RNA loads compared to the heart (*p* < 0.05), brain (*p* < 0.05), and testis (*p* < 0.01). Similarly, the serum showed lower YFV RNA loads than the testis (*p* < 0.05). Additionally, in females, the liver had significantly lower YFV RNA loads than the brain (*p* < 0.05) (Figure 4). These results indicate that YFV RNA loads were significantly higher in the brain, testis, heart, and thymus, compared to liver and serum from 8 to 14 dpi [female from 8 dpi (n = 2) and 14 dpi (n = 3); male from 8 dpi (n = 1), 11 dpi (n = 2), and 14 dpi (n = 2)] (Figure 4).

**Figure 5:**
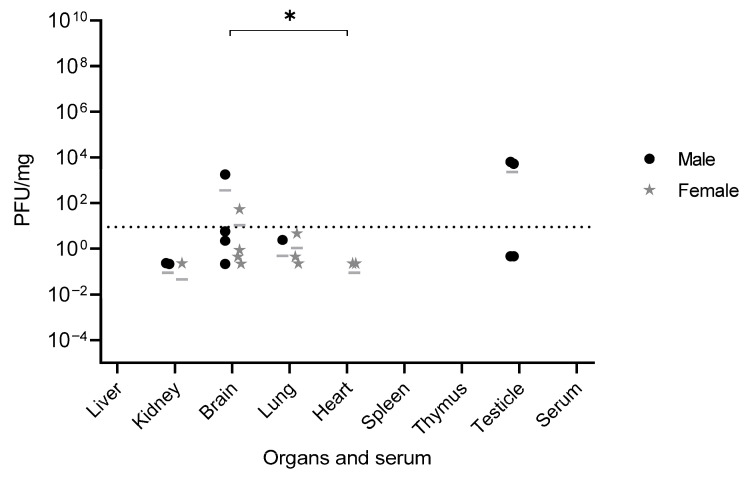
Infectious viral load in serum and various organs of four-week-old C57BL/6 IFNAR1^−/−^ mice infected with wild-type YFV_HS306/2018. Male and female mice (n = 5 per group) were infected subcutaneously with 5.75 × 10^3^ PFU of YFV_HS306/2018. Following euthanasia, blood, brain, eye, thymus, heart, lungs, liver, spleen, kidneys, and testis were collected and subjected to viral titration by plaque assay. Results are presented as mean values. Statistical analysis was performed using the Kruskal–Wallis test. Statistically significant difference was observed between brain vs. heart. Significance level: *p* < 0.05 (*). Dashed line = assay’s sensitivity threshold. PFU = plaque-forming unit.

Infectious viral titers were assessed in serum, brain, thymus, heart, lungs, liver, spleen, kidneys, and testis by plaque assay, using the same post-euthanasia samples tested for YFV RNA loads. Infectious viral particles were detected in the testes, brain, kidneys, heart, and lungs. In contrast, no viral particles were detected in the liver, spleen, thymus, or serum. Moreover, titers in the kidneys, heart, and lungs were below the assay’s sensitivity threshold of 20 PFU per well [19]. Samples, such as serum, thymus, liver, and spleen, had no infectious viral particles detected. There were no statistically significant differences in infectious viral loads between male and female organs, or between the organs of survivors and euthanized animals. However, the brain harbored significantly higher infectious viral loads than the heart (*p* < 0.05) (Figure 5).

## 4. Discussion

In this study, four-week-old male and female C57BL/6 IFNAR1^−/−^ mice were successfully infected with WT YFV_HS306/2018, belonging to a new lineage isolated during the 2018 outbreak in Brazil, and they developed disease consistent with infection by YFV. Taken together, our findings are consistent with those of previous studies [20,21,22,23,24], which reported clinical signs characteristic of viscerotropic disease, as piloerection, hunched posture, and swelling at the inoculation site, similar to those observed in our model. In addition, these studies show that both the route of infection and viral strain influence the pathogenesis of YFV in mice. Notably, the clinical progression, timing of symptom onset, and mortality observed in our study closely resembled those reported by Meier et al. (2009) [20], suggesting that infection with WT YFV via the subcutaneous route produces comparable disease in IFNAR1^−/−^ mice across different genetic backgrounds.

C57BL/6 IFNAR1^−/−^ mice infected with WT YFV from 2018, presented 60% survival for the female mice and 40% for the male mice. In contrast, the study conducted by Meier et al. (2009) [20] demonstrated that A129 IFNAR1^−/−^ mice infected with 1.0 × 10^4^ PFU Asibi and Angola73 strains had 100% lethality, and euthanasia was performed between 7 and 8 dpi [20]. Additionally, C57BL/6 STAT2^−/−^ mice infected with 10^4^ PFU of YFV-Asibi experienced 100% survival until the study endpoint on day 6 dpi [21].

Most published studies to date have used the YFV-17D vaccine strain to investigate YF pathogenesis in mice. Studies using 3–4-week-old C57BL/6 PVR-Tg21 IFNAR1^−/−^ mice, as well as in 2-week-old A129 mice, have shown both viscerotropic and neurotropic disease, with lethality reaching 100% [22,23,24]. Intraplantar infection of four-week-old A129 mice, with YFV-17D, led to limited viral replication at the inoculation site only [24], in contrast to the broader viral dissemination observed in this study using a WT YFV strain.

Infectious particles of YFV_HS306/2018 were detected in the testis, brain, kidneys, heart, and lungs, some of them associated with virus tropism observed in humans, such as the heart, kidneys [13,14] and brain [25]. These findings correlate with the pathogenesis and the clinical signs observed in infected mice including penile inflammation, tremors, and moribund state, highlighting the tropism and pathogenesis of WT YFV. Other studies have also detected viral presence in similar organs, related to the pathogenesis of YFV. For instance, in a study using YFV-17D via intramuscular injection, viral RNA was found in the spleen on day 1 and in the muscle, sciatic nerve, blood, liver, spleen, and brain on day 3 dpi. However, by days 7 and 8 dpi, the virus was undetectable in blood and liver [22], consistent with our observations of undetectable infectious particles in serum, liver, spleen, and thymus at later stages of infection, likely reflecting advanced disease progression.

The detection of YFV_HS306/2018 RNA in serum, brain, eye, thymus, heart, lungs, liver, spleen, kidneys, and testis, and infectious particles in testis, brain, kidneys, heart, and lungs, provides insight into YFV_HS306/2018 organ tropism. Earlier time points analyzed in other studies also show the tropism of YFV for peripheral organs and the brain. In subcutaneous infections with YFV WT, viral replication was detected in draining lymph nodes early after infection, with higher titers in spleen, liver, bone marrow, and brain at 6 dpi, the final time point assessed [20]. In intramuscular infection models with YFV WT, viral RNA was detected in the liver and spleen at 3 and 6 dpi [21]. Following intraperitoneal infection, bioluminescent imaging revealed viral presence in the brain, heart, liver, lungs, spleen, kidneys, intestine, and testis by 5 dpi [24]. Based on our findings and previous studies [20,21,24], it is possible to observe that the detection of the YFV in different organs is closely related to the time point of the organ collection, with replication sites varying along the infection progression in time.

One limitation of our study is the small sample size per experimental group, which prevented temporal analysis of viral load across different days post-infection. In that way, earlier stages of infection could not be assessed due to sample limitations. Also, samples from animals that succumbed at different dpi and those that survived to the study endpoint (14 dpi) had to be analyzed together. However, the findings highlight the relevance of the animal model and support its potential for investigating YFV pathogenesis and tissue tropism in future studies.

An important and novel finding of this study is the detection of WT YFV_HS306/2018 in the testes following peripheral infection. Viral RNA was detected in the testis of all infected mice, both those euthanized at 8 and 11 dpi and those that survived to 14 dpi. Infectious viral particles were also recovered from the testis of 4 out of 5 infected animals. Furthermore, the magnitude of viral replication in the testes was remarkable. The genomic viral load in testicular tissue and in the brain was significantly higher than that observed in the liver and serum. These results are consistent with those reported in A129 IFNAR1^−/−^ mice infected with ZIKV, in which the highest ZIKV RNA levels were found in the testes, brain, and spleen compared to other organs [26]. Notably, detectable levels of infectious viruses were observed only in convalescent mice, suggesting that viral replication in the testes occurs later in infection. Other studies have similarly reported delayed detection of YFV in various organs. For example, infected individuals have shown late-onset hepatitis persisting for months after symptom onset, with YFV RNA and antigen detectable in liver biopsies [27,28]. Prolonged viral RNA shedding in urine has also been observed for over a month after symptom onset, suggesting sustained viral replication in the kidneys [29]. While the presence of WT YFV HS306/2018 was demonstrated here, we could not test if similar outcomes occur with other WT YFV strains commonly used in experimental studies, such as Asibi and Angola73, under the same conditions. Such comparative experiments would be critical to determine whether testicular infection is a unique property of this genotype or a broader feature of YFV biology. Therefore, this limitation should be acknowledged, and future research should address whether other WT YFV strains display similar testicular tropism following peripheral infection in mice.

This work represents the first to investigate sex-related differences in the course of YFV infection in four-week-old C57BL/6 IFNAR1^−/−^ mice. Under the experimental conditions used, no significant differences were observed between males and females in susceptibility to YFV. A clinical study by Kallas et al. (2019) [30] evaluated naturally infected individuals during the 2018 outbreak in São Paulo and included 68 male and 8 female patients with YF. In their multivariate analysis, only age emerged as an independent predictor of mortality [30]. However, observational studies during outbreaks often suffer from sampling bias, as males tend to be more frequently exposed and diagnosed at different stages of infection. Therefore, animal models are valuable tools for evaluating sex-related differences in disease progression under controlled conditions. Given the lack of studies specifically addressing sex-based differences in yellow fever, further research using diverse animal models and viral lineages is warranted.

In summary, this study demonstrates that both male and female C57BL/6 IFNAR1^−/−^ mice develop comparable clinical manifestations following WT YFV_HS306/2018 infection, with minor differences in recovery time. Viral RNA was broadly distributed across multiple organs in both sexes, consistent with YFV viral tropism observed in humans and nonhuman primates, and infectious particles were recovered from different tissues, including the brain and testis. Notably, this is the first report to identify infectious wild-type YFV in testicular tissue. These findings collectively support the establishment of the C57BL/6 IFNAR1^−/−^ mouse as a viable model for YFV infection, offer important insights into the tissue tropism, and contribute to the biological characterization of YFV_HS306/2018, a newly isolated Brazilian strain.

## Data Availability

Raw data is available at Zenodo under https://doi.org/10.5281/zenodo.16995776.

## Supplementary Materials

The following supporting information can be downloaded at: https://www.mdpi.com/article/10.3390/v17101325/s1, Figure S1: Clinical signs observed in four-week-old C57BL/6 IFNAR1^−/−^ mice infected with WT YFV; Table S1: Scoring of the clinical signs observed in four-week-old C57BL/6 IFNAR1^−/−^ mice infected with WT YFV.

## Abbreviations

The following abbreviations are used in this manuscript:

dpi	Days post-infection
FBS	Fetal bovine serum
IFN	Interferon
IFNAR	Interferon receptor
IVL	Infectious viral load
MEM	Minimum essential medium
NHP	Non-human primates
PBS	Phosphate-buffered saline
PFU	Plaque-forming unit
RNA	ribonucleic acid
RNAL	RNA load
RT-qPCR	Reverse transcription quantitative polymerase chain reaction
UTR	Untranslated region
WT	Wild type
YF	Yellow fever
YFV	Yellow fever virus
ZIKV	Zika virus
