# Supplementary material for: In Vivo Characterization and Tissue Tropism of a Wild-Type Yellow Fever Virus Isolate from the 2017–2018 Brazilian Outbreak in C57BL/6 IFNAR1−/− Mice
Source: Viruses. 2025 Sep 29;17(10):1325. doi: 10.3390/v17101325 (PMC12567742; doi:10.3390/v17101325)
Supplement: Supplementary file 1 [file viruses-17-01325-s001.zip › viruses-3776857-supplementary.pdf]

Characterization of a Novel Wild-Type Yellow Fever Virus Isolate from the 2017–2018 Brazilian Outbreak – Part II – In vivo studies: biological characterization and tissue tropism inC57BL/6 IFNAR1$^{-/-}$ mice. Cruz et al.

Supplementary Materials

Table S1: Scoring of the clinical signs used to observe four-week-old C57BL/6 IFNAR1$^{-/-}$ mice infected with WT YFV.

ID	Score	Category	Clinical sign
A	0		Healthy status
B	1	Mild	Inoculation site inflammation
	2		Mild piloerection
C	3		Moderate piloerection
	4		Severe piloerection
	5		Mild hunched back
D	6		Moderate hunched back
	7		Severe hunched back
E	8	Moderate	Swelling on the face
F	9		Conjunctivitis
G	10		Penis inflammation
H	11		Tremors
I	12	Severe	Bleeding
J	13		Moribund

Each parameter was assigned an identification (ID) using a letter (A to J) to facilitate data entry into a spreadsheet during the experiments. Each parameter has an associated score, which correlates with the severity (category) of the clinical signs. Scores were adapted from LAZEAR et al, 2016 [17] and adapted.

Clinical sign	Male											Female										
	Animal	Dpi										Animal	Dpi									
		5	6	7	8	9	10	11	12	13	14		5	6	7	8	9	10	11	12	13	14
J																						
I																						
H														11								
G				10	10																	
F	♂ 1		9	9		9	9	9	9	9	9	♀ 1		9								
E				8		8	8	8	8		5											
D			5	6	6	6	6	6	5	5	2			6	6	5	6	5	5	5		
C			2	3	3	2	2	2	2	2				3	4	2	3	2	2	2		
B		1	1	1									1	1	1							
A																					0	0
J					13											13						
I																12						
H					11																	
G					10																	
F	♂ 2											♀ 2			9	9						
E				8	8										8	8						
D			5	6	7									5	7	7						
C			2	3	4									2	4	4						
B		1	1	1									1	1	1							
A																						
J								13														
I																						
H					11	11		11														
G				10	10	10	10	10														
F	♂ 3		9	9				9				♀ 3		9			9					
E			8	8	8	8	8	8						8								
D			6	7	7	7	7	7						5	6	5	5			5		
C			3	3	3	3	3	4						2	3	2	2			2		
B		1	1	1									1	1	1							
A																		0	0		0	0
J								13														
I																						
H															11		11	11				
G																						
F	♂ 4							9				♀ 4			9	9	9					
E			8	8	8	8	8	8							8							
D			5	6	7	7	7	7						5	7	7	6	5	5	5		
C			2	3	4	3	3	4						2	4	3	3	2	2	2		
B		1	1	1									1	1	1							
A																					0	0
J																	13					
I																						
H			11	11																		
G																						
F	♂ 5											♀ 5		9	9	9	9					
E				8											8	8						
D			5	6	6	5	5	5	5		5			5	7	7	7					
C			2	3	3	2	2	2	2		2			2	4	4	4					
B		1	1	1									1	1	1							
A										0												

Figure S1: Clinical signs observed in four-weeks-old C57BL/6 IFNAR1-/- mice infected with WT YFV. Male and female mice (n=5 per group) were inoculated with 5.75 x 10^3 PFU of YFV_HS306/2018 and monitored daily for clinical signs. The mock groups were inoculated with supernatants from uninfected cells. Each clinical sign was assigned an identification (ID) with a letter (A to J) to facilitate data entry into a spreadsheet during the experiments. Each parameter has a corresponding score related to the severity of the clinical signs (A-D: mild; E-H: moderate; I-J: severe), detailed in table S1. The clinical signs shown here are from day 5 post-infection (dpi), which is when they first became observable. Dpi = days post infection. Grey: euthanized mice.
